# Impact of Prior Abdominal Surgery on Rates of Conversion to Open Surgery and Short-Term Outcomes after Laparoscopic Surgery for Colorectal Cancer

**DOI:** 10.1371/journal.pone.0134058

**Published:** 2015-07-24

**Authors:** Ik Yong Kim, Bo Ra Kim, Young Wan Kim

**Affiliations:** 1 Department of Surgery, Division of Gastrointestinal Surgery, Yonsei University Wonju College of Medicine, Wonju, Korea; 2 Department of Internal Medicine, Division of Gastroenterology, Yonsei University Wonju College of Medicine, Wonju, Korea; Heinrich-Heine-University and University Hospital Duesseldorf, GERMANY

## Abstract

**Purpose:**

To evaluate the impact of prior abdominal surgery (PAS) on rates of conversion to open surgery and short-term outcomes after laparoscopic surgery for colon and rectal cancers.

**Methods:**

We compared three groups as follows: colon cancer patients with no PAS (n = 272), major PAS (n = 24), and minor PAS (n = 33), and rectal cancer patients with no PAS (n = 282), major PAS (n=16), and minor PAS (n = 26).

**Results:**

In patients with colon and rectal cancers, the rate of conversion to open surgery was significantly higher in the major PAS group (25% and 25%) compared with the no PAS group (8.1% and 8.9%), while the conversion rate was similar between the no PAS and minor PAS groups (15.2% and 15.4%). The 30-day complication rate did not differ among the three groups (28.7% and 29.1% in the no PAS group, 29.2% and 25% in the major PAS group, and 27.3% and 26.9% in the minor PAS group). The mean operative time did not differ among the three groups (188 min and 227 min in the no PAS group, 191 min and 210 min in the major PAS group, and 192 min and 248 min in the minor PAS group). The rate of conversion to open surgery was significantly higher in patients with prior gastrectomy or colectomy compared with the no PAS group, while the conversion rate was similar between the no PAS group and patients with prior radical hysterectomy in patients with colon and rectal cancers.

**Conclusions:**

Our results suggest that colorectal cancer patients with minor PAS or patients with prior radical hysterectomy can be effectively managed with a laparoscopic approach. In addition, laparoscopy can be selected as the primary surgical approach even in patients with major PAS (prior gastrectomy or colectomy) given the assumption of a higher conversion rate.

## Introduction

Despite accumulating evidence for the short-term clinical advantages of laparoscopic colorectal surgery and its oncologic equivalence to open procedures [1–4], this minimally invasive surgical approach has not been widely adopted in patients with a history of prior abdominal surgery (PAS). The main reasons are technical difficulties in adhesiolysis and prolonged operative time [5]. A previous abdominal operation inevitably results in intra-abdominal adhesions, and the ergonomic constraints of laparoscopic instruments make it difficult to achieve sufficient adhesiolysis. In addition, laparoscopic adhesiolysis increases the risk of inadvertent bowel injury [6,7].

To date, a history of PAS is no longer considered a relative contraindication for laparoscopy; however, PAS is likely to increase the complexity of the procedure [8]. There are conflicting data available with regard to laparoscopic colorectal surgery in patients with PAS. Law et al. [9] reported that a history of PAS did not negatively influence short-term clinical outcomes with respect to postoperative complication rate, ileus, open conversion rate, and duration of hospital stay after laparoscopic colorectal surgery. In contrast, Yamamoto et al. [10] showed higher rates of intraoperative enterotomy and postoperative ileus, and delayed time to diet in patients with PAS.

We hypothesized that laparoscopy for colorectal cancer is feasible and safe for selected patients with PAS, and specific types of PAS may guide preoperative patient selection for laparoscopic colorectal cancer resection. To date, there are studies comparing outcomes between patients with and without PAS after laparoscopic surgery for benign and malignant colorectal diseases [5,9–16]. The current study included only malignant conditions and compared the conversion rates and short-term outcomes of the three groups as follows: patients with no PAS, those with major PAS, and those with minor PAS undergoing laparoscopy for colon and rectal cancer.

## Methods

### Patients

This retrospective cohort study was conducted at a tertiary referral center. All clinical investigations were conducted according to the principles expressed in the Declaration of Helsinki. Informed consent was obtained from all patients before surgery. All participants provided written informed consent and the ethics committee (the institutional review board of Wonju Severance Christian Hospital) approved this retrospective study (YWMR-155013). All patient records/information was de-identified prior to analysis. This observational study was conducted using the STROBE (STrengthening the Reporting of OBservational studies in Epidemiology) guidelines [17]. Eligibility criteria included having histologically confirmed colorectal cancer and undergoing major laparoscopic colorectal resection between March 1, 2008 and February 28, 2014. Patients undergoing palliative non-resectional or bypass surgery, emergent operation, trans-anal local excision, or multi-visceral resection were not eligible for this study. Among 653 patients, 99 patients (15.2%) had a history of any type of abdominal surgery. Among these, 40 patients (6.1%) had major PAS. We compared conversion rates and short-term outcomes among the three groups as follows: colon cancer patients with no PAS (n = 272), major PAS (n = 24), and minor PAS (n = 33) undergoing laparoscopy for colon cancer, and rectal cancer patients with no PAS (n = 282), major PAS (n = 16), and minor PAS (n = 26) undergoing laparoscopy for rectal cancer.

### Classification of prior abdominal surgery

Prior abdominal surgery (PAS) was defined as any previous abdominal surgery by either laparoscopy or laparotomy, and was classified as major and minor PAS. Major PAS was defined as abdominal surgery involving more than one abdominal quadrant accessed through midline incision from the xiphoid to the umbilicus or from the umbilicus to the symphysis pubis. Minor PAS was defined as abdominal surgery involving one abdominal quadrant through a right subcostal, paramedian, Rockey-Davis or Pfannenstiel incision. Low midline incisions for minor or benign gynecologic surgery, such as oophorectomy, salphingectomy, Cesarean section, or abdominal hysterectomy for myoma, were classified as minor PAS. Laparoscopic abdominal surgeries, such as cholecystectomy or appendectomy, were classified as minor PAS.

### Study endpoints

The primary endpoint was to investigate the rate of conversion to open surgery after laparoscopic surgery for colon and rectal cancer among patients without PAS, with minor PAS, and with major PAS. Secondary endpoints were to evaluate short-term outcomes (operative time, inadvertent enterotomy, postoperative complication rate, hospital stay, and time to diet) after laparoscopic surgery for colon and rectal cancer among patients without PAS, with minor PAS, and with major PAS.

### Surgery, adjuvant therapy and follow-up

All operations were performed by two experienced colorectal surgeons. One surgeon had over 700 cases of laparoscopic experience, and the other had over 200 cases of laparoscopic experience. Before surgery, the type of surgical approach—laparoscopy or open procedure—was discussed with patients and their families. Patients with right colon cancer did not receive any mechanical bowel preparation and fasted only from midnight the night before the surgery. Patients with left colon and rectal cancers received mechanical bowel preparation with 4 L of polyethylene glycol solution. A glycerin enema was performed twice, once in the afternoon and once in the evening on the day before the surgery. First-generation cephalosporin (cefazolin) was used as a prophylactic antibiotic and was administered just before the start of surgery. Postoperative antibiotic treatment was continued for 24 to 48 hours. After standardized preoperative preparation, complete mesocolic excision with central vascular ligation was performed in patients with colon cancer. Tumor-specific mesorectal excision including high ligation of the inferior mesenteric artery was performed for rectal cancer. The operative principles underlying the laparoscopic approach were basically the same as those of complete mesocolic excision or tumor-specific mesorectal excision through laparotomy. After recovery from surgery, chemotherapy was recommended for patients with stage II, III and IV disease according to National Comprehensive Cancer Network (NCCN) guidelines. All patients were registered in an electronic colorectal database and were followed until death or December 31, 2014. Median follow-up period was 45.6 months (interquartile range, 21.8 to 67.2 months).

### Outcome measures

All laparoscopy data were analyzed according to the intention-to-treat principle. Postoperative complications were defined as events that required additional treatment within 30 days of surgery. Wound infection was defined according to the definition of the Centers for Disease Control (CDC) as superficial or deep incisional surgical site infection occurring in the surgical wound [18]. All postoperative events were graded based on the Clavien-Dindo classification [19]. Conversion to open surgery was defined as stopping the laparoscopic approach and using a conventional laparotomy incision to complete the planned surgical procedure.

### Statistical analysis

All statistical analyses were performed using MedCalc Statistical Software version 15.2.2 (MedCalc Software bvba, Ostend, Belgium) and IBM SPSS Statistics for Windows, version 22.0 (IBM, Armonk, NY, USA). The Student’s t-test was used for comparison of continuous variables, and the Chi-squared test (or Fisher's exact test) was used for comparison of categorical variables. A p-value <0.05 was considered to be statistically significant.

### Sample size calculation

Sample size calculation was performed using PASS 2008 ver. 8.0 (NCSS, LLC, Kaysville, UT, USA). Arteaga Gonzalez et al.[12] reported conversion rates in patients with (26.1%) or without prior abdominal surgery (5.1%, p = .02) after laparoscopic colorectal surgery. Thus, our sample size was calculated using these results. Group sample sizes of 41 in no PAS group and 41 in the PAS group two achieve 80% power to detect a difference between the group proportions of .2100. The proportion in group one (the PAS group) is assumed to be .0510 under the null hypothesis and .2610 under the alternative hypothesis. The proportion in group two (the no PAS group) is .0510. For the test statistic, we used the two-sided Z test with pooled variance. The significance level of the test was targeted at .0500. The significance level actually achieved by this design was .0456. The final sample size was 41 patients in each group. We finally enrolled 57 colon cancer patients with PAS and 42 rectal cancer patients with PAS.

## Results

### Patient characteristics

Baseline characteristics of the patients in the 3 groups are presented in **Table 1**. The major PAS (66.7%) and minor PAS (69.7%) groups had a significantly higher proportion of female patients compared to the no PAS group (39.3%) among patients with colon cancer. Although not significant, the major PAS (43.8%) and minor PAS (42.3%) groups had a higher proportion of female patients compared to the no PAS group (27.7%) in patients with rectal cancer. Age, body mass index, American Society of Anesthesiologists (ASA) score, and the incidence of pT4 tumor did not differ among the three groups.

**Table 1:** Patient characteristics.

**Colon cancer (n = 329)**	No PAS (n = 272)	Major PAS (n = 24)		Minor PAS (n = 33)	
	N (%), mean (SD)	N(%), mean(SD)	p*	N(%), mean(SD)	p^†^
Age (years)	68(11.1)	67(12.0)	.644	64(12.3)	.052
Sex (female)	107(39.3)	16(66.7)	.009	23(69.7)	.001
Body mass index (kg/m^2^)	23.9(3.2)	23.4(3.7)	.483	23.9(3.5)	.978
ASA score (≥3)	44(16.2)	1(4.2)	.116	2(6.1)	.125
pT4 (+)	48(17.6)	2(8.3)	.243	10(30.3)	.080
Operation					
Right colectomy	163(59.9)	11(45.8)	.090	13(39.4)	.020
Sigmoid, left colectomy	99(36.4)	10(41.7)		20(60.6)	
Other	10(3.7)	3(12.5)		0(.0)	
**Rectal cancer (n = 324)**	No PAS (n = 282)	Major PAS (n = 16)		Minor PAS (n = 26)	
	N(%), mean(SD)	N(%), mean(SD)	p*	N(%), mean(SD)	p^†^
Age (years)	68(10.8)	67(10.3)	.904	68(9.0)	.978
Sex (female)	78(27.7)	7(43.8)	.166	11(42.3)	.115
Body mass index (kg/m^2^)	23.7(3.5)	22.8(4.5)	.359	25(2.8)	.067
ASA score (≥3)	34(12.1)	2(12.5)	.958	4(15.4)	.622
pT4 (+)	21(7.4)	3(18.8)	.106	1(3.8)	.495
Preoperative radiation (+)	73(25.9)	3(18.8)	.524	8(30.8)	.588
Operation					
Abdominoperineal resection	35(12.4)	0(.0)	.035	2(7.7)	.703
Low anterior resection	245(86.9)	15(93.8)		24(92.3)	
Other	2(.7)	1(6.3)		0(.0)	

PAS, prior abdominal surgery; SD, standard deviation; ASA, American Society of Anesthesiologists

* Comparison between the no PAS and major PAS groups.

^†^ Comparison between the no PAS and minor PAS groups.

### Reasons for prior abdominal surgery

The detailed reasons for PAS are presented in **Table 2**. In colon cancer patients, radical hysterectomy with extended lymph node dissection for gynecologic cancer was most common (41.7%) in the major PAS group, followed by left colectomy and proctectomy (16.7%), and gastrectomy (8.3%). In the minor PAS group, appendectomy was most common (45.5%), followed by gynecologic procedures (27.3%). In rectal cancer patients, gastrectomy was most common (43.8%), followed by radical hysterectomy with extended lymph node dissection for gynecologic cancer (18.8%). In the minor PAS group, appendectomy was most common (50.0%), followed by gynecologic procedures (7.7%).

**Table 2:** Reasons for prior abdominal surgery.

**Colon cancer (n = 57)**			
Major PAS (n = 24)	N(%)	Minor PAS (n = 33)	N(%)
Gynecologic surgery (radical hysterectomy with extended lymph node dissection)*	10(41.7)	Appendectomy	15(45.5)
Gastrectomy	2(8.3)	Gynecologic surgery	9(27.3)
Left colectomy, proctectomy	4(16.7)	Cholecystectomy	6(18.2)
Right colectomy	3(12.5)	Other	3(9.1)
Small bowel resection	2(8.3)		
Urologic surgery (bladder, kidney)	1(4.2)		
Hepatobiliary surgery	2(8.4)		
**Rectal cancer (n = 42)**			
Major PAS (n = 16)	N(%)	Minor PAS (n = 26)	N(%)
Gynecologic surgery (radical hysterectomy with extended lymph node dissection)*	3(18.8)	Appendectomy	13(50.0)
Gastrectomy	7(43.8)	Gynecologic surgery	2(7.7)
Left colectomy	1(6.3)	Cholecystectomy	3(11.5)
Right colectomy	2(12.5)	Stoma creation	1(3.8)
Small bowel resection	2(12.5)	Other	7(26.9)
Urologic surgery (bladder)	1(6.3)		

PAS, prior abdominal surgery

* Cesarean section (n = 4), abdominal hysterectomy for uterine myoma (n = 2), and laparoscopic gynecologic procedures (n = 5) were excluded.

### Short-term outcomes of colon cancer patients

In patients with colon cancer, the rate of conversion to open surgery was significantly higher in the major PAS group (25%) compared with the no PAS group (8.1%), while the conversion rate was similar between the no PAS and minor PAS groups (15.2%). Adhesion-related conversion was more common in the major PAS (50%) and minor PAS (20%) groups when compared to no PAS group (9.1%). The 30-day complication rate did not differ among the three groups (28.7% in the no PAS group, 29.2% in the major PAS group, and 27.3% in the minor PAS group). The wound infection rate did not differ among the three groups (8.1% in the no PAS group, 8.3% in the major PAS group, and 9.1% in the minor PAS group). Mean operative time did not differ among the three groups (188 min in the no PAS group, 191 min in the major PAS group, and 192 min in the minor PAS group). The intraoperative enterotomy rate did not differ among the three groups (2.6% in the no PAS group, 8.3% in the major PAS group, and 3.0% in the minor PAS group). Other intraoperative and postoperative outcomes such as blood loss, mortality, severity of complications (Clavien-Dindo grade), time to soft diet, hospital stay, and time to chemotherapy initiation did not differ among the groups.

Pathologic results with respect to distal margin, number of lymph nodes retrieved, and tumor size were all comparable in the major and minor PAS groups compared with the no PAS group. When comparing TNM classification, stage IV disease was more common in the minor PAS group (18.2%) compared with the no PAS group (3.7%) (**Table 3**).

**Table 3:** Short-term outcomes of colon cancer patients (n = 329).

		No PAS (n = 272)	Major PAS (n = 24)		Minor PAS (n = 33)	
		N(%), mean(SD)	N(%), mean(SD)	p*	N(%), mean(SD)	p^†^
Conversion to open surgery(+)		22(8.1)	6(25.0)	.007	5(15.2)	.177
Adhesion-related		2(9.1)	3(50.0)		1(20.0)	
Tumor fixation (locally advanced tumor)		17(77.3)	3(50.0)		3(60.0)	
Technical reason (bleeding)		3(13.6)	0(.0)		1(20.0)	
30-day complication rate (+)		78(28.7)	7(29.2)	.959	9(27.3)	.866
Wound infection		22(8.1)	2(8.3)		3(9.1)	
Anastomotic leakage		8(2.9)	1(4.2)		1(3.0)	
Postoperative ileus		15(5.5)	0(.0)		1(3.0)	
Operative time (min)		188(62)	191(66)	.827	192(55)	.752
Median		178	180		190	
Range		69, 450	105, 345		95, 310	
Interquartile range		145, 220	138, 235		148, 225	
Blood loss (ml)		106(209)	100(140)	.877	83(84)	.535
Intraoperative enterotomy (+)		7(2.6)	2(8.3)	.115	1(3.0)	.877
30-day mortality (+)		2(.7)	0(.0)	.673	0(.0)	.621
Clavien-Dindo score (≥3)		23(8.5)	2(8.3)	.983	4(12.1)	.484
Mean time to soft diet (days)		6(3)	7(7)	.295	6(2)	.730
Mean hospital stay (days)		12(7)	12(10)	.799	12(5)	.873
Time to chemotherapy (days)		33.1(9)	32.8(4)	.924	30.6(5)	.263
TNM classification	0, I	69(25.4)	8(33.3)	.071	8(24.2)	.003
	II	88(32.4)	11(45.8)		6(18.2)	
	III	105(38.6)	3(12.5)		13(39.4)	
	IV	10(3.7)	2(8.3)		6(18.2)	
Lymph node harvest (number)		23(11)	21(12)	.566	27(13)	.052
Distal margin (cm)		11.8(10)	12.7(9)	.663	15.3(9)	.063
Tumor size (cm)		4.6(2)	3.9(2)	.113	4.2(2)	.178

PAS, prior abdominal surgery; SD, standard deviation.

* Comparison between the no PAS and major PAS groups.

^†^ Comparison between the no PAS and minor PAS groups

### Short-term outcomes of rectal cancer patients

In patients with rectal cancer, the rate of conversion to open surgery was significantly higher in the major PAS group (25%) compared with the no PAS group (8.9%), while the conversion rate was similar between the no PAS and minor PAS groups (15.4%). Adhesion-related conversion was more common in the major PAS (50%) and minor PAS (25%) groups, when compared to no PAS group (8%). The 30-day complication rate was 29.1% in the no PAS group, 25% in the major PAS group, and 26.9% in the minor PAS group, respectively. The wound infection rate was 4.3% in the no PAS group, 6.3% in the major PAS group, and 11.5% in the minor PAS group, respectively. Mean operative time was 227 min in the no PAS group, 210 min in the major PAS group, and 248 min in the minor PAS group, respectively. The intraoperative enterotomy rate was 2.1% in the no PAS group, 6.3% in the major PAS group, and 3.8% in the minor PAS group, respectively. Other intraoperative and postoperative outcomes such as blood loss, mortality, severity of complications (Clavien-Dindo grade), time to soft diet, hospital stay, and time to chemotherapy initiation did not differ among the groups (**Table 4**).

**Table 4:** Short-term outcomes of rectal cancer patients (n = 324).

		No PAS (n = 282)	Major PAS (n = 16)		Minor PAS (n = 26)	
		N(%), mean(SD)	N(%), mean(SD)	p*	N(%), mean(SD)	p^†^
Conversion to open surgery(+)		25(8.9)	4(25.0)	.034	4(15.4)	.276
Adhesion-related		2(8.0)	2(50.0)		1(25.0)	
Tumor fixation (locally advanced tumor)		20(80.0)	2(50.0)		2(50.0)	
Technical reason (bleeding)		3(12.0)	0(.0)		1(25.0)	
30-day complication rate (+)		82(29.1)	4(25.0)	.726	7(26.9)	.817
Wound infection		12(4.3)	1(6.3)		3(11.5)	
Anastomotic leakage		15(5.3)	1(6.3)		1(3.8)	
Postoperative ileus		16(5.7)	1(6.3)		2(7.7)	
Operative time (min)		227(84)	210(60)	.414	248(75)	.236
Median		213	195		235	
Range		90, 625	135, 360		130, 420	
Interquartile range		170, 270	163, 245		205, 300	
Blood loss (ml)		174(274)	89(115)	.233	233(305)	.313
Intraoperative enterotomy (+)		6(2.1)	1(6.3)	.290	1(3.8)	.574
30-day mortality (+)		1(.4)	0(.0)	.811	0(.0)	.761
Clavien-Dindo score (≥3)		47(16.7)	2(12.5)	.662	3(11.5)	.497
Mean time to soft diet (days)		5(4)	6(6)	.637	5(3)	.551
Mean hospital stay (days)		14(11)	13(9)	.590	13(8)	.593
Time to chemotherapy (days)		37.3(15)	48.6(50)	.518	50.9(49)	.282
TNM classification	0, I	84(29.8)	7(43.8)	.452	8(30.8)	.714
	II	83(29.4)	3(18.8)		5(19.2)	
	III	97(34.4)	6(37.5)		11(42.3)	
	IV	18(6.4)	0(.0)		2(7.7)	
Circumferential margin (+)		26(9.3)	2(12.5)	.669	0(.0)	.104
Lymph node harvest (number)		18(9)	18(7)	.839	17(7)	.507
Distal margin (cm)		3.2(3)	2.7(2)	.510	3.1(5)	.859
Tumor size (cm)		4(2)	3.5(2)	.373	3.4(2)	.160

PAS, prior abdominal surgery; SD, standard deviation.

* Comparison between the no PAS and major PAS groups.

^†^ Comparison between the no PAS and minor PAS groups

### Outcomes according to types of major prior abdominal surgery

In colon cancer patients, outcomes were compared between the no PAS group and patients with prior gastrectomy or colectomy (n = 9), and patients with prior radical hysterectomy with extended lymph node dissection for gynecologic cancer (n = 10). The rate of conversion to open surgery was significantly higher in patients with prior gastrectomy or colectomy (33.3%) compared with the no PAS group (8.1%), while the conversion rate was similar between the no PAS group and patients with prior radical hysterectomy (20.0%). There were no differences among the three groups with respect to 30-day complication rates (28.7% in the no PAS group, 11.1% in patients with prior gastrectomy or colectomy, and 40% in patients with radical hysterectomy) and operative time (188 min in the no PAS group, 203 min in patients with prior gastrectomy or colectomy, and 191 min in patients with radical hysterectomy). Other postoperative outcomes such as time to diet, duration of hospital stay, and lymph node harvest were similar among the three groups.

In patients with rectal cancer, outcomes were compared between the no PAS group and patients with prior gastrectomy or colectomy (n = 10), and patients with prior radical hysterectomy with extended lymph node dissection for gynecologic cancer (n = 3). The rate of conversion to open surgery was significantly higher in patients with prior gastrectomy or colectomy (30.0%) compared with the no PAS group (8.9%), while the conversion rate was similar between the no PAS and patients with prior radical hysterectomy (0.0%). There were no differences among the three groups with respect to 30-day complication rates (29.1% in the no PAS group, 20.0% in patients with prior gastrectomy or colectomy, and 33.3% in patients with radical hysterectomy) and operative time (227 min in the no PAS group, 199 min in patients with prior gastrectomy or colectomy, and 200 min in patients with radical hysterectomy). Other postoperative outcomes such as time to diet, duration of hospital stay, and lymph node harvest were similar among the three groups (**Table 5**).

**Table 5:** Outcomes according to type of major prior abdominal surgery.

**Colon cancer**	No PAS (n = 272)	Prior gastrectomy or colectomy (n = 9)		Prior gynecologic surgery (n = 10)	
	N(%), mean(SD)	N(%), mean(SD)	p*	N(%), mean(SD)	p^†^
Age (years)	68(11)	73(6)	.041	60.8(12)	.040
Sex (female)	107(39.3)	4(44.4)	.758	10(100.0)	< .001
Conversion to open surgery	22(8.1)	3(33.3)	.009	2(20.0)	.185
30-day complication rate (+)	78(28.7)	1(11.1)	.249	4(40.0)	.439
Operative time (min)	188(62)	203(83)	.508	191(58)	.884
Time to soft diet (days)	6(3)	5(2)	.599	5.1(2)	.572
Hospital stay (days)	12(7)	9(2)	.327	11.1(3)	.772
Lymph node harvest (number)	23(11)	19(14)	.424	22(12)	.888
**Rectal cancer**	No PAS (n = 282)	Prior gastrectomy or colectomy (n = 10)		Prior gynecologic surgery (n = 3)	
Age (years)	68(11)	68(10)	.979	66(14)	.786
Sex (female)	78(27.7)	3(30.0)	.871	3(100.0)	.006
Conversion to open surgery	25(8.9)	3(30.0)	.026	0(.0)	.589
30-day complication rate (+)	82(29.1)	2(20.0)	.533	1(33.3)	.872
Operative time (min)	227(84)	199(65)	.282	200(57)	.573
Time to soft diet (days)	5(4)	7(8)	.343	4(2)	.585
Hospital stay (days)	14(11)	15(11)	.974	10(3)	.507
Lymph node harvest (number)	18(9)	17(8)	.696	20(8)	.784

PAS, prior abdominal surgery; SD, standard deviation

* Comparison between the no PAS and major PAS groups (patients with prior gastrectomy or colectomy)

^†^ Comparison between the no PAS and major PAS groups (patients with prior gynecologic surgery)

### Outcomes of converted patients with major prior abdominal surgery

In colon cancer patients, outcomes were compared between the no PAS and major PAS groups who completed laparoscopy (n = 18) and those who underwent conversion to laparotomy (n = 6). The 30-day complication rate, operative time, time to diet, and duration of hospital stay did not differ among the groups. Intraoperative enterotomy was more frequent in non-converted patients with major PAS (11.1%) than in patients with no PAS (2.6%, p = 0.043).

In rectal cancer patients, outcomes were compared between the no PAS and major PAS groups who completed laparoscopy (n = 12) and those who underwent conversion to laparotomy (n = 4). Thirty-day complication rate, operative time, intraoperative enterotomy time to diet, and duration of hospital stay did not differ among the groups (**Table 6**).

**Table 6:** Outcomes of converted patients with major prior abdominal surgery.

**Colon cancer**	No PAS (n = 272)	Converted patients with major PAS (n = 6)		Non-converted patients with major PAS (n = 18)	
	N(%), mean(SD)	N(%), mean(SD)	p*	N(%), mean(SD)	p^†^
30-day complication rate (+)	78(28.7)	3(50.0)	.256	4(22.2)	.556
Operative time (min)	188(62)	237(73)	.063	176(58)	.421
Intraoperative enterotomy (+)	7(2.6)	0(.0)	.691	2(11.1)	.043
Time to soft diet (days)	6(3)	6(2)	.712	7(8)	.639
Hospital stay (days)	12(7)	12(3)	.902	12(12)	.820
Lymph node harvest (number)	23(11)	14(7)	.076	23(13)	.749
**Rectal cancer**	No PAS (n = 282)	Converted patients with major PAS (n = 4)		Non-converted patients with major PAS (n = 12)	
30-day complication rate (+)	82(29.1)	1(25.0)	.858	3(25.0)	.760
Operative time (min)	227(84)	259(73)	.460	194(49)	.169
Intraoperative enterotomy (+)	6(2.1)	0(.0)	.768	1(8.3)	.167
Time to soft diet (days)	5(4)	5(1)	.528	6(7)	.372
Hospital stay (days)	14(11)	16(10)	.745	12(9)	.417
Lymph node harvest (number)	18(9)	15(7)	.467	19(8)	.858

* Comparison between the no PAS and converted patients of major PAS groups

^†^ Comparison between the no PAS and non-converted patients of major PAS groups

## Discussion

The major findings of this study were that a higher rate of conversion to open surgery was observed in the major PAS group compared with the no PAS group in patients with colon cancer and rectal cancers, respectively. Specific types of major PAS (prior gastrectomy or colectomy) increased the conversion rates in patients with colon and rectal cancers, respectively. However, the converted patients with major PAS did not have worse clinical outcomes in terms of operative time, postoperative complication rate, and time to diet. Between the no PAS and major PAS groups, other short-term outcomes did not differ. In addition, all short-term outcomes in the minor PAS group were comparable to those in the no PAS group.

In patients with previous abdominal operations, intra-abdominal adhesions are troublesome and must be cleared for oncologic resection. Moreover, it is difficult to predict the severity and degree of adhesions preoperatively, complicating patient selection for a laparoscopic approach in patients with PAS. Thus, we categorized types of PAS to predict preoperative suitability for laparoscopy. Data on prior operations showed that gynecologic surgery was most common in the major PAS group in colon cancer patients and gastrectomy was most common in the major PAS group in rectal cancer patients, while appendectomy and gynecologic surgery were most common in the minor PAS group. When compared with the no PAS group, there were more women in the major and minor PAS groups, likely due to the high incidence of gynecologic surgery. In the present study, conversion rates and short-term outcomes did not differ between the no PAS and minor PAS groups. Minor PAS (abdominal surgical procedures involving one quadrant of the abdominal cavity) is unlikely to cause widespread adhesions or influence the clinical outcomes of subsequent surgery [20]. Thus, colorectal cancer patients with minor PAS may be safely managed with a laparoscopic approach.

In the current study, conversion to open surgery was more frequent in the major PAS group compared to the no PAS group. Furthermore, adhesion-related conversion was more common in the major PAS group (50% in colon cancer and 50% in rectal cancer, respectively) when compared to the no PAS group (9.1% in colon cancer and 8% in rectal cancer) and minor PAS group (20% in colon cancer and 25% in rectal cancer). Arteaga Gonzalez et al. [12] reported that a history of prior surgery was associated with higher conversion rates (26.1% vs. 5.1%), and Franko et al. [13] also observed a higher rate of conversion in the prior surgery group (19.6% vs. 11.4%). However, other investigators did not observe this difference in conversion rate [5,9–11,15,20]. We observed that the conversion rate was not different between the no PAS and minor PAS groups. In colon and rectal cancer patients, the most common reason for conversion was tumor fixation due to locally advanced tumor in the no PAS and minor PAS groups followed by technical issues such as uncontrolled bleeding. Thus, the higher rate of conversion in the major PAS group may be attributed to resultant adhesions. Patient-related factors (obesity or adhesion) and tumor-related factors (locally advanced or bulky tumors) are well-known risk factors for conversion to open surgery [1,3,21]. Similarly, we observed that a history of major PAS was a significant risk factor for conversion. Specifically, higher conversion rates were observed in patients with prior gastrectomy or colectomy, but not in patients with prior radical hysterectomy with extended lymph node dissection for gynecologic cancer.

We also evaluated outcomes of converted patients with major PAS because several studies reported adverse outcomes in converted patients, including higher morbidity, increased transfusion, longer operative time, prolonged hospital stay [14,22,23]. In our study, short-term outcomes were not worse in converted patients. Operative time, intraoperative enterotomy, 30-day complication rate, time to diet, and duration of hospital stay were the same in converted and laparoscopic patients. In colon and rectal cancer patients with major PAS, converted patients had a lengthy operative time when compared to patients with no PAS or non-converted patients with major PAS. However, these differences did not reach statistical significance due to the small sample size. We believe that appropriate surgical decision-making regarding conversion to an open approach is important for patient safety and oncologic results during laparoscopic procedures. Timely conversion to open surgery may avoid prolonged operative time or prevent unnecessary tumor manipulation and tumor cell spillage, thus reducing the potential risk of adverse clinical and oncologic outcomes [1,24]. Indeed, Belizon et al. [22] suggested that prompt conversion within 30 minutes of the start of surgery may reduce adverse postoperative outcomes. Unfortunately, we could not assess the time point of conversion to open surgery due to the retrospective nature of data collection. Thus, conversion should be regarded as another strategy to solve the technical difficulties of laparoscopy for colorectal cancer.

On review of literature, short-term outcomes in patients with PAS are variable. Beck et al. [25] demonstrated that previous abdominal surgery significantly prolongs time to opening the abdomen. Vignali et al. [5] reported longer operative times in patients with PAS (218 min vs. 192 min) in their case-matched study. However, other investigators observed similar operative times between patients with PAS and those without, as was observed in our series [9–12,15,20]. Subsequent surgery in patients with major PAS increases operative time because adhesiolysis in patients who have been operated on in four quadrants can take a substantial amount of time. In the current study, although statistically nonsignificant, patients with major PAS had shorter operative times. A test for normality with the Shapiro-Wilk method was used to determine whether operative time data were normally distributed. Although the sample size was small, operative time data showed a normal distribution pattern: colon cancer patients with major PAS (p = .087) and minor PAS (p = .348), and rectal cancer patients with major PAS (p = .258) and minor PAS (p = .383). There could be two reasons for shorter operative times in patients with major PAS in this study. First, operative time is mainly affected by surgical procedure type, and, accordingly, differences among the no PAS, major PAS, and minor PAS groups probably influence operative time. In colon cancer patients, right colectomy was more frequently performed in the no PAS group when compared to major PAS and minor PAS groups. At our institution, right colectomy for right-sided colon cancer was routinely performed based on complete mesocolic excision with central vascular ligation, which generally necessitates a lengthy operative time. In rectal cancer patients, abdominoperineal resection was commonly performed in the no PAS group when compared to major PAS and minor PAS groups. Operative time for abdominoperineal resection is generally longer than that for low anterior resection due to perineal dissection. Second, to reduce operative time in patients with major PAS, adhesions irrelevant to the planned surgical procedures were not removed.

A higher rate of inadvertent enterotomy has been reported in patients with a history of prior surgery [10,13]; however, PAS did not influence risk of inadvertent enterotomy in this study and others [20]. In the literature, the intraoperative enterotomy rate ranges from 0.1 to 0.2% in the no PAS group and 0.9 to 5.5% in the PAS group [10,13,26]. The rather high rate of enterotomy in this study was partly due to inclusion of colon and rectal cancer patients. Unlike laparoscopy for benign colorectal diseases, laparoscopic surgery for colorectal cancer focuses on oncologic clearance and radicality. Laparoscopic complete mesocolic excision for colon cancer or laparoscopic total mesorectal excision for rectal cancer are procedures with increased technical complexity. Franko et al. [13] observed a higher incidence of postoperative ileus and reoperation in patients with PAS, and Aytac et al. [20] reported a higher postoperative complication rate in the PAS group. However, we observed similar postoperative complication rates in the major and minor PAS groups compared with the no PAS group [5,9–12,15]. Yamamoto et al. observed that PAS increased time to diet significantly, but most other studies, including ours, reported that PAS did not increase time to diet [9,12]. In this study, hospital stay was similar in the PAS groups compared with the no PAS group. In previous literature, PAS did not influence length of hospital stay [5,9–12,15,20]. Favorable short-term outcomes of the major and minor PAS groups are in line with other published data [9,11,12,15]. We believe that sufficient surgeon experience and surgical team training positively influenced these findings [27]. Rates of wound infection after laparoscopic colorectal surgery have been reported to range from 4 to 12% [4,28,29]; wound infection rates in our subgroups varied from 4.3 to 11.5%, which is within this reported range.

This study was limited by its retrospective nature and the relatively small number of patients included in a single center. We included only malignant cases, categorized types of PAS such as minor and major, and evaluated the impact of types of PAS on outcomes after laparoscopic surgery for colon and rectal cancers. The incidence of PAS ranges from 21 to 46% in the literature [5,9–13,15] and the incidence in this study was 15%. The low rate of prior surgery may be related to the retrospective nature of the data collection and the possibility that the patient's surgical history was missed in the history taking before surgery. In addition, surgeon preference might have influenced the choice of laparoscopy for patients with previous history of abdominal surgery.

In summary, if technical difficulties are anticipated during surgery, surgeons tend to decline a laparoscopic approach [30]. Our results suggest that colorectal cancer patients with minor PAS or patients with prior radical hysterectomy with extended lymph node dissection for gynecologic cancer can be effectively managed with a laparoscopic approach. Specific types of major PAS (prior gastrectomy or colectomy) increased conversion rates in patients with colon and rectal cancers. The higher rate of conversion observed in the major PAS group was partly due to the underlying adhesions related to major PAS. However, other short-term outcomes of the major PAS group are comparable to the no PAS group. Moreover, short-term outcomes were not worse in the converted patients with major PAS. Thus, laparoscopy can be selected as the primary surgical approach even in patients with major PAS (prior gastrectomy or colectomy) given the assumption of a higher conversion rate. In the future, it would be helpful to formulate a management pathway according to types of PAS.
